# Electrically Coded Retinomorphic Spectrophotodetector

**DOI:** 10.1002/adma.74160

**Published:** 2026-07-17

**Authors:** Mohit Kumar, Hyunmin Dang, Hyungtak Seo

**Affiliations:** ^1^ Department of Energy Systems Research Ajou University Suwon Republic of Korea; ^2^ Department of Materials Science and Engineering Ajou University Suwon Republic of Korea

**Keywords:** color classification, in‐sensor, retinomorphic, self‐powered, sub‐nanometer

## Abstract

Accurate color sensing is essential for applications ranging from materials analysis to autonomous vision, yet compact systems that can classify color directly at the device level without bulky optics or computationally intensive post‐processing remain limited. Here, a machine learning‐free, self‐powered retinomorphic pyro‐photodetector is demonstrated for direct electrical wavelength encoding from 365 to 940 nm. The multiterminal Ag/ZnO/*n*‐Si/Ag device uses electrostatically balanced built‐in potentials to convert incident wavelength into distinct photo and pyroelectric current fingerprints, enabling direct in‐sensor spectral discrimination without spectral reconstruction and external neural processing. The device achieves wavelength decoding with less than 3 nm accuracy and a pyroelectric current rise‐time of ∼46 µs. At the system level, direct in‐sensor encoding enables end‐to‐end wavelength classification with 300 ms latency while reducing downstream energy and computational complexity. The encoded electrical readout enables accurate color classification and is further applied to plant health and pigment‐state sensing, as well as quantification of 0% to 40% water adulteration in milk, curd, coconut water, and salt and sugar solutions, with classification accuracy above 92%. This work establishes a portable retinomorphic platform for real‐time, energy‐efficient, and high‐precision spectral sensing, providing a general route toward direct in‐sensor color recognition and wavelength classification.

## Graphical abstract

Self‐powered retinomorphic pyro‐photodetector is demonstrated that avoids machine‐learning post‐processing and covers 365–940 nm. Electrostatic balancing of built‐in potential produces an electrical wavelength code, delivering <3 nm wavelength decoding accuracy with ∼46 µs response. The encoded signal enables color discrimination, plant‐pigment, and health‐assessment, and quantifies 0%–40% water adulteration in milk, curd, coconut water, and salt/sugar solutions with >92% accuracy.

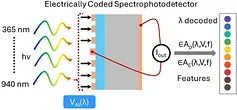

## 1 Introduction

**FIGURE 1:**
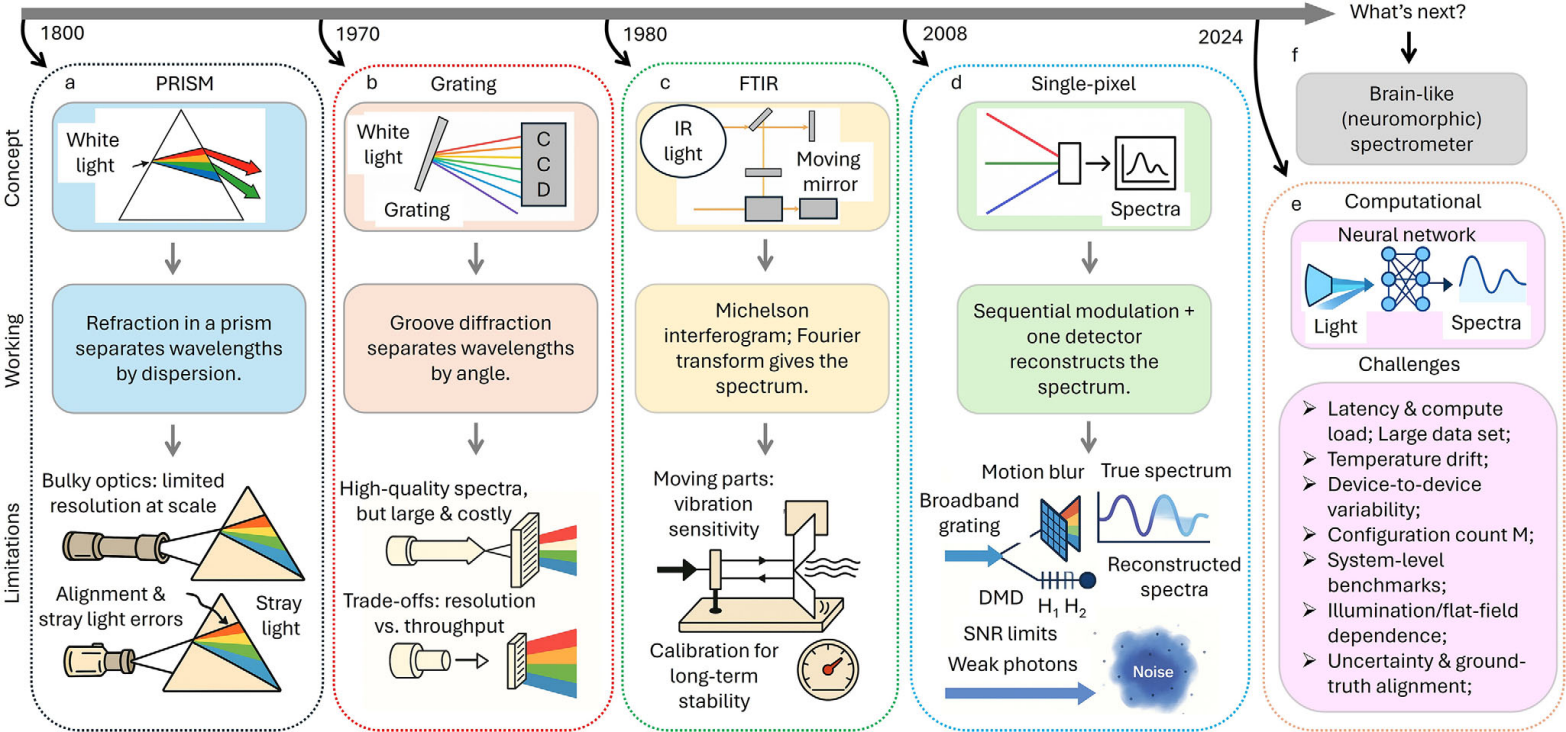
From dispersion to neuromorphic spectrometry. (a), (b) Dispersion spectrometers (prism, grating+CCD) scale in size and inherit resolution–etendue trade‐offs. (c) FTIR achieves broadband fidelity but relies on moving elements and long‐term calibration. (d) Single‐pixel/compressive systems reconstruct spectra from sequential patterns, with SNR/motion limits. (e) Computational spectrometers learn an inverse mapping but incur latency/compute, calibration drift, and large configuration counts. (f) This work: a neuromorphic spectrometer that uses pyro‐phototronic, event‐driven signals to output task variables directly without spectral reconstruction, reducing configurations, energy, and latency.

Accurate color sensing underpins a wide range of scientific and technological applications—from materials characterization and chemical diagnostics to remote sensing and autonomous vision [1, 2, 3]. Conventional spectrometers—prism, grating, and interferometric—achieve fidelity by physically separating wavelengths; however, the long optical paths, alignment tolerances, and stray‐light sensitivity constrain compact, robust deployment (Figure 1a,b) [4, 5, 6]. Interferometric instruments extend bandwidth by encoding spectra into interferograms and retrieving them via Fourier transforms (i.e., Fourier transform infrared, FTIR) yet moving components and the need for long‐term calibration limit suitability for fieldable and real‐time applications (Figure 1c) [7]

To overcome these limitations, compact computational‐optics spectrometers avoid spatial dispersion and instead encode spectra into multiplexed intensity patterns that are numerically inverted (Figure 1d) [8]. In particular, miniaturized architectures such as single‐pixel/compressive designs, metasurface, and disordered‐medium encoders markedly shrink optics; but the burden shifts to calibration and stability: fabrication variability perturbs the encoding matrix, temperature and illumination changes induce drift, and motion or low‐photon regimes depress signal‐to‐noise ratio [9, 10, 11]. Thus, while these approaches reduce size and complexity, usable accuracy in practice often depends on frequent recalibration and tightly controlled conditions—trading size gains for operational fragility.

More recently, machine‐learning‐assisted spectrometers attempt to bypass optical complexity by training neural networks to reconstruct spectra, in short to classify colors directly from compressed detector outputs (Figure 1e) [9, 10, 11, 12, 13, 14, 15, 16, 17]. This flexibility is appealing for diverse tasks, yet it introduces system‐level costs: acquiring radiometrically reliable datasets, preserving generalization across devices and environments, and meeting latency and energy budgets on edge platforms [15, 16, 17, 18]. When illumination, temperature, and aging shifts occur, models commonly require adaptation, and the compute/memory footprint can become the limiting resource. Consequently, real‐time, low‐power operation remains elusive and device‐to‐device generalization is often weak. These limitations motivate a different strategy: treat spectral sensing as a decision problem rather than a reconstruction problem. A neuromorphic perspective emphasizes event‐driven transduction and compact, fixed stimulus sets that generate low‐dimensional yet robust signatures for immediate classification (Figure 1f). Indeed, human color vision is based on the comparative responses of photoreceptors with different spectral sensitivities, followed by neural processing of the relative response pattern [19]. Thus, biological color perception relies on encoded differential responses rather than direct spectral reconstruction [20]. In the same functional spirit, retinomorphic device does not reconstruct the full spectrum through bulky optics and intensive digital processing. Instead, it converts the incident wavelength into a compact electrical fingerprint through wavelength‐dependent photoresponse and pyroelectric amplitudes across multiple bias programmed channels. By minimizing configuration complexity and off‐sensor computation, such front‐ends prioritize the quantities that matter in real applications—stability, latency, and energy efficiency—without relying on bulky dispersive optics or continual retraining.

Here, we develop and experimentally demonstrate a machine‐learning‐free, self‐powered (0 V), broadband UV–visible–near‐infrared (365–940 nm) multiterminal pyro‐photodetector in which multiple alternating bias frequencies encode wavelength as a joint photo‐ and pyro‐current amplitude fingerprint, enabling hardware‐level in‐sensor feature encoding with simultaneous temporal and spectral discrimination. By coupling optical absorption, charge transport, and electrostatic modulation within a single element—via carefully balanced built‐in potentials—our device performs purely electrical wavelength encoding with <3 nm wavelength decoding accuracy and a ∼46 µs pyroelectric current rise time, and uses this physically encoded readout to realize accurate color classification, plant health and pigment‐state sensing, and quantification of water adulteration (0%–40%) in milk, curd, coconut water, and salt/sugar solutions with >92% accuracy. This paradigm of on‐chip, real‐time, energy‐efficient, and drift‐tolerant color recognition overcomes longstanding bulk‐optics trade‐offs and the computational overheads of machine learning, positioning our portable retinomorphic photodetector as a general platform for nanometer‐precision spectral sensing across science, industry, and healthcare.

Recent miniaturized spectral sensing studies have shown that compact spectral identification can be achieved in electrically tunable van der Waals platforms. Yoon et al. [13] demonstrated accurate wavelength identification using a single tunable van der Waals junction, and Cui et al. [21] further extended this concept to broadband spectral sensing for material identification. However, those approaches rely on electrically tuned optoelectronic responses in van der Waals heterojunctions [13, 21]. The novelty of the present work lies in the direct electrical encoding of wavelength information within a self‐powered multiterminal Ag/ZnO/*n*‐Si/Ag retinomorphic pyro‐photodetector, in which frequency multiplexed lateral bias modulation generates a characteristic joint photo and pyroelectric electrical fingerprint for each incident wavelength directly within the sensing element itself. In this device, wavelength dependent built‐in field redistribution, pyroelectric transients, and electrostatically balanced built‐in potentials act together with multifrequency lateral biasing to produce a hardware level retinomorphic encoding scheme for direct wavelength classification. Unlike conventional spectrometers that rely on optical dispersion, and unlike machine learning assisted approaches that depend on digital spectral reconstruction or trained classification models, the present device performs wavelength identification through physically encoded in sensor signal generation. This mechanism also distinguishes the present platform from conventional ZnO/Si photodetectors and earlier pyro phototronic devices, which have focused mainly on signal enhancement rather than spectral fingerprint generation and direct classification (see Table S1, Supporting Information).

## 2 Results and Discussion

**FIGURE 2:**
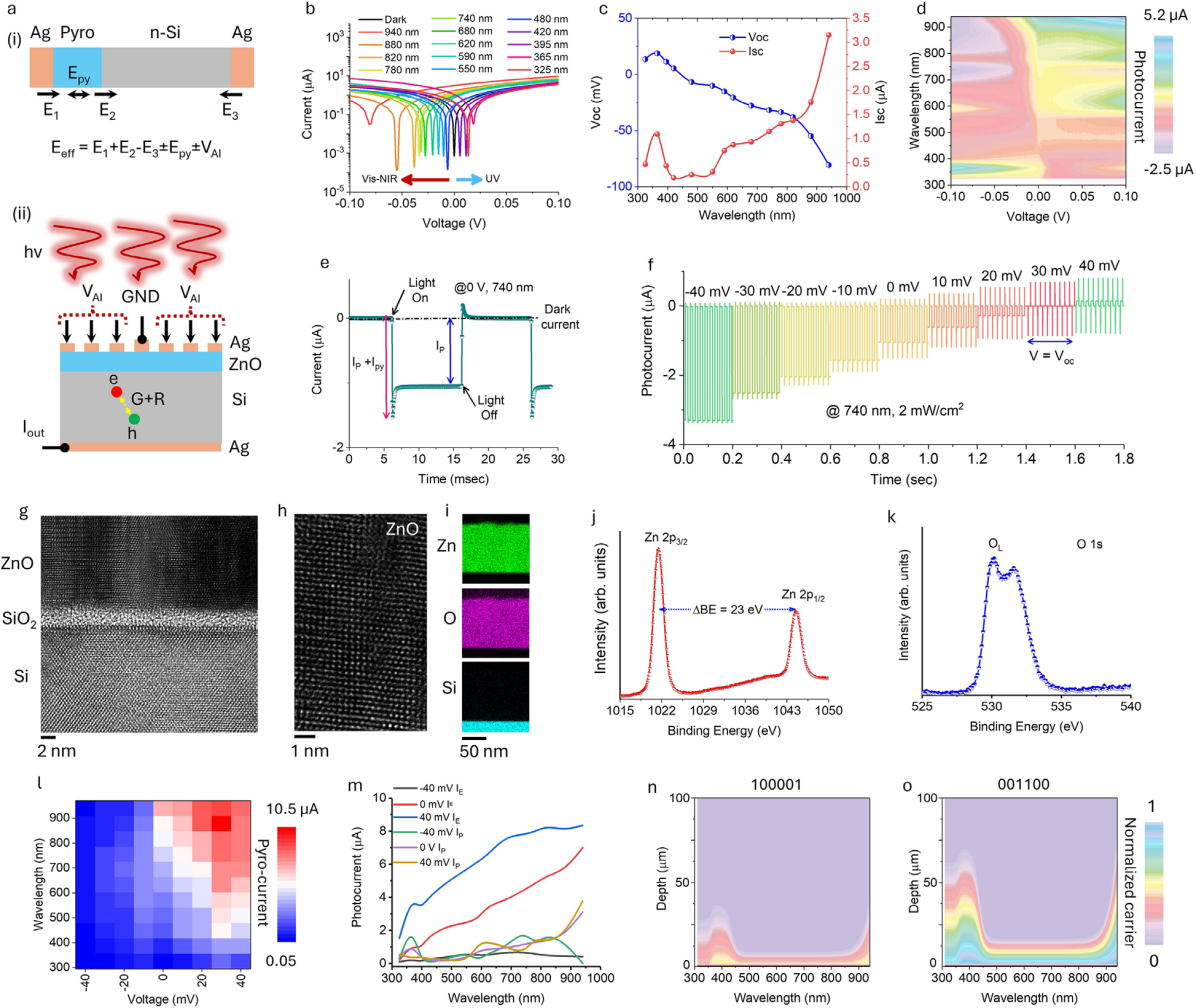
Pyro‐phototronic multi‐terminal ZnO/Si photodetector and basic device characteristics. (a) Schematic of the Ag/pyroelectric/n‐Si/Ag stack. (i) Three metal/semiconductor junctions generate built‐in fields *E*_1_, *E*_2_, and *E*_3_; together with the pyroelectric field *E*_py_and the lateral analog‐inference bias *V*_AI_, they define the effective driving field *E*_eff_ = *E*_1_ + *E*_2_ − *E*_3_ ± *E*_py_ ± *V*_AI_. (ii) Multi‐terminal operation: an array of Ag top electrodes on ZnO is biased with programmable *V*_AI_ around a grounded reference while pulsed illumination generates photo–pyro carriers in the ZnO/Si region that are collected as an output current *I*_out_ at the bottom Ag contact. (b) Semi‐logarithmic *I*–*V* characteristics measured from –0.10 to +0.10 V in the dark and under monochromatic illumination (325–940 nm), showing a wavelength‐dependent polarity inversion between UV and Vis–NIR with arrows. (c) Extracted open‐circuit voltage *V*_oc_ (left axis) and short‐circuit current *I*_sc_ (right axis) versus wavelength, highlighting a sign change of *V*_oc_ across the UV–visible boundary and a broadband increase of ∣*I*_sc_∣ toward the NIR. (d) Two‐dimensional map of photocurrent as a function of wavelength and bias. (e) Transient response at 740 nm and 0 V: sharp negative and positive spikes at light on/off (pyroelectric component *I*_py_) superimposed on a quasi‐steady photocurrent plateau *I*_P_. (f) Bias‐dependent mixed photo–pyro response at 740 nm for external voltages from –40 to +40 mV. At *V* ≈ *V*_oc_ the DC component vanishes but strong bipolar spikes persist. g) and h) Cross‐sectional high‐resolution TEM image of the ZnO/SiO_x_/Si stack in which the amorphous interfacial SiOx layer is approximately 2.5 nm thick, and enlarged view of the ZnO layer, showing dense, crystalline films and an ultrathin interfacial oxide. i) STEM–EDS elemental maps of Zn, O, and Si confirming a well‐defined ZnO layer on Si. (j), (k) XPS spectra of the ZnO film: Zn 2*p* core levels (j) with a spin–orbit splitting ΔBE ≈ 23 eV characteristic of Zn^2+^ in ZnO, and O 1s (k) showing a dominant lattice‐oxygen peak. (l) Map of photo‐pyro current amplitude versus wavelength and bias, demonstrating that this can also be programmed by small voltages. (m) Spectral dependence of steady photocurrent *I*_P_ and event current *I*_E_ at selected biases, illustrating independent, bias‐tunable weighting of the quasi‐static and I_E_. (n), (o) simulated depth–wavelength distributions of normalized collected carriers in the ZnO/Si stack under two representative lateral‐bias patterns (e.g., “100001” and “001100”), showing how bias reconfigures the internal collection landscape and underpins the device's electrically coded spectral selectivity.

Following our approach, we fabricate a pyroelectric photodetector by depositing a ∼100 nm ZnO layer on *n*‐Si using atomic layer deposition (Experimental section and Supporting Information, Figure S1). Figure 2a(i) schematically summarizes the possible internal (build‐in) fields in the Ag/pyroelectric/*n*‐Si/Ag device. The three metal/semiconductor junctions generate built‐in potentials *E*_1_, *E*_2_, and *E*_3_ with different polarities across the structure, and their vector sum, together with the pyroelectric field *E*_py_ and externally applied lateral bias *V*_AI_, defines the effective driving field: *E*_eff_ = *E*_1_ + *E*_2_ − *E*_3_ ± *E*_py_ ± *V*_AI_. Here *V*_AI_ (external analog inference voltage) is a programmable control knob that modulates both the steady‐state photocurrent and the pyroelectric spike amplitude and thereby acts as an in‐sensor feature extractor [22, 23, 24, 25]. Importantly, the Ag/pyroelectric (Ag/ZnO) contact is most active in the ultraviolet regime, where absorption in the ZnO layer dominates, whereas for visible to near‐infrared photons the response is mainly governed by the ZnO/Si and Si/Ag junctions inside the silicon [26]. In contrast to these wavelength‐dependent built‐in fields, the pyroelectric potential arises from instant changes in light intensity and therefore contributes to carrier separation over the entire spectral range, with a magnitude and sign that depends on wavelength.

Figure 2a(ii) illustrates the multi‐terminal operation, in which Ag top electrodes (separated 2 mm) are biased with programmable lateral voltages *V*_AI_ around a grounded reference contact. Under pulsed photo illumination (*h*ν), the combined action of the junction built‐in fields, pyroelectric potential, and lateral bias shapes the internal potential landscape, modulates the local generation–recombination (*G* + *R*) dynamics in the ZnO/Si region, and directs the resulting photo–pyro charge separation toward the bottom Ag contact, where the output current *I*_out_ is collected. By appropriately choosing the spatial pattern and amplitude of *V*_AI_ (six here in number but not limited to) the device physically encodes high‐level features—such as wavelength and temporal modulation—into a small set of analog photo‐pyro current signatures. In contrast to conventional machine‐learning pipelines, where digital feature extraction dominates the computational cost, our architecture performs this step directly in the sensor hardware. As a result, the downstream classifier can be reduced to simple, low‐energy operations, enabling—in principle—up to ∼90 % reduction in overall energy consumption for a given classification task and providing a general framework for in‐sensor feature extraction [27].

Figure 2b shows the semi‐logarithmic current–voltage characteristics of the Ag/ZnO/n‐Si/Ag device measured from –0.10 to +0.10 V in the dark and under monochromatic illumination from 325 to 940 nm. In the dark, the current around zero bias remains below ∼10^−3^ µA, confirming low leakage. Under illumination, the zero‐bias current increases into the ∼µA range depending on wavelength—an enhancement of approximately three orders of magnitude relative to the dark state. A pronounced polarity inversion of the photocurrent appears as the excitation is swept from the UV to the visible–NIR region: for UV wavelengths (325–395 nm) the I–V curves cross the origin with positive current at small positive bias, whereas for visible to NIR wavelengths (≥480 nm) the current at the same bias becomes negative. This sign reversal reflects the shift in the dominant junction: in the UV regime, absorption is concentrated near the top Ag/ZnO contact so that this junction dictates the net built‐in field, while for longer wavelengths photogeneration occurs mainly in the silicon and the ZnO/Si and Si/Ag junctions govern carrier separation and thus set the current polarity. Similar wavelength‐dependent flipping of the polarity of open‐circuit voltage (*V*_oc_) has been reported previously in a single device [26]. The I‐V characteristics in Figure 2b were measured by a standard voltage sweep under continuous monochromatic illumination and therefore represent the steady‐state photocurrent response of the Ag/ZnO/*n*‐Si/Ag device rather than the transient pyroelectric response. Here, the ZnO/Si junction should be understood more precisely as a ZnO/SiO_x_/Si interface containing an ultrathin native interlayer. The strong wavelength dependence of the I‐V characteristics originates from the wavelength‐dependent redistribution of the effective internal field in the device, because ultraviolet absorption is dominated near the Ag/ZnO junction, whereas visible and near infrared photogeneration occurs mainly in silicon and shifts the dominant carrier separation pathway to the ZnO/Si and Si/Ag junctions.

The extracted photovoltaic parameters are presented in Figure 2c. The *V*_oc_ is positive (∼+30–40 mV) in the UV (325–365 nm), decreases with increasing wavelength, and crosses zero near the UV–visible boundary (around 420 nm). For visible and NIR illumination, *V*_oc_ becomes negative, reaching approximately –40 to –60 mV between 550 and 800 nm and up to ∼–80 to –90 mV at 940 nm. In contrast, the magnitude of short‐circuit current (*I*_sc_) remains finite over the entire 325–940 nm range: it is on the order of 0.3 µA at 325 nm, exhibits a local maximum of ∼0.7–1 µA around 365–395 nm, shows a shallow minimum (∼0.1 µA) near 500 nm, and then increases monotonically toward the NIR, reaching ∼1.5 µA at 780–880 nm and ∼3 µA at 940 nm. The combination of a wavelength‐dependent sign changes in *V*_oc_ with a broadband, gradually increasing ∣*I*_sc_∣demonstrates that the device reconfigures its internal field landscape with wavelength: the Ag/ZnO junction dominates in the UV, whereas the ZnO/Si and Si/Ag junctions control the response in the visible–NIR. This built‐in, polarity‐encoded evolution of *V*_oc_ and *I*_sc_ provides an intrinsic spectral fingerprint of the incident light that can later be exploited for in‐sensor classification [28].

For clarity, Figure 2d represents the wavelength‐dependent I–V characteristics into a two‐dimensional photocurrent map as a function of voltage (–0.10 to +0.10 V, horizontal axis) and wavelength (300–940 nm, vertical axis). The color scale encodes the photocurrent from approximately –2.5 to +5.2 µA, so that warm colors correspond to negative current and cool colors to positive current. Around zero bias, the map clearly shows a transition from positive photocurrent in the UV (∼325–365 nm) to negative photocurrent in the visible–NIR regime (≥480 nm), in agreement with the polarity inversion discussed for Figure 2b,c. This polarity boundary appears as a contour that shifts slightly with wavelength and applied bias, reflecting the gradual rebalancing of the contributions from the Ag/ZnO, ZnO/Si, and Si/Ag junctions to the net internal field. In the UV, a portion of high positive current extends over a broad range of small forward and reverse biases, indicating that the Ag/ZnO junction efficiently separates photocarriers generated near the top contact [22]. At longer wavelengths, the dominant response moves toward negative current, and its magnitude increases toward the NIR, consistent with deeper absorption in silicon and carrier collection governed by the ZnO/Si and Si/Ag interfaces. Overall, this compact 2D map visualizes how the device continuously reconfigures its internal potential landscape with both wavelength and bias, providing a rich, polarity‐encoded photocurrent signature that can be directly exploited as an analog feature space for in‐sensor classification.

Figure 2e resolves the transient response of the device at 740 nm under zero external bias. In the dark, the current remains low, which is dark current [29, 30]. When the light is switched on, the current exhibits a sharp negative spike with an amplitude of about –1.5 µA, which then relaxes within a few milliseconds to a quasi‐steady photocurrent plateau of ∼–0.8 µA. This initial “overshoot” corresponds to the combined contribution of the steady photocurrent and the pyroelectric current (*I*_P_ + *I*_py_), whereas the long‐lived plateau represents the pure photocurrent (*I*_P_) driven by the built‐in fields [24, 31, 32]. When the light is turned off, an opposite‐polarity spike of about –0.2 µA appears and decays back to the dark level, consistent with a pyroelectric response triggered by the rapid decrease in optical intensity. These measurements confirm that, even at 0 V, the device simultaneously supports a quasi‐static photovoltaic component and a fast, bipolar pyroelectric component superimposed on it (Figures S2 and S3, Supporting Information). The time required for the transient current to reach its peak after light switching was approximately 46 µs under near‐open‐circuit operation. Under a small reverse bias, the corresponding rise time was ∼150 µs (Figure S4, Supporting Information). This ∼46 µs value refers to the intrinsic device transient response, namely the pyroelectric current rise time under abrupt light switching and should not be interpreted as the end‐to‐end classification time of the full sensing system.

The bias dependence of the mixed photo–pyro response is depicted in Figure 2f, which shows the pulsed photocurrent at 740 nm (intensity = 2 mW cm^−2^) while the external bias is stepped from –40 to +40 mV in 10 mV increments. At the most negative bias (–40 mV) the pulses are strongly negative, with magnitude ∼3.2 µA and relatively small spike amplitudes. As the bias is swept from –40 mV toward 0 mV, both photocurrent and the spike height change: the average current becomes progressively less negative and the amplitude of the pyroelectric spikes is gradually changing, although clear on/off transients remain at every bias. When the bias is further increased into forward direction (10–20 mV), the pulse train shifts upward and eventually oscillate around a small net current. In the range around +30–40 mV, indicated as V ≈ V_oc_, the average photocurrent over one pulse period is close to zero while distinct pyroelectric spikes persist [33, 34]. This indicates that, by applying an external bias, we can compensate for the internal built‐in potential; however, spike generation is linked to the instantaneous change in illumination intensity, so the pyro‐current still spikes rapidly. Thus, by adjusting only a few tens of millivolts, the device can be tuned continuously from a strongly reverse‐biased photovoltaic regime to a nearly zero‐DC operating point, without losing the spike‐like pyroelectric transients that are crucial for event‐driven sensing and spike‐based feature extraction [33, 34]. Because the illumination was continuous during this measurement, no pulse duration is associated with Figure 2b, and the pyroelectric contribution becomes significant only under abrupt temporal changes in illumination, as demonstrated by the spike‐like transients in Figure 2e,f.

Because the pyroelectric current in ZnO is closely linked to its crystalline structure, we first examined the crystallinity of the ZnO film by TEM. Figure 2g–i confirm the structural quality and compositional sharpness of the ZnO/Si heterostructure used in our devices [22, 24, 33]. The cross‐sectional high‐resolution TEM image in Figure 2g shows the ZnO layer grown on crystalline Si, separated by an ultrathin native SiOx interlayer. From the HR TEM image, the thickness of this amorphous interfacial layer is estimated to be approximately 2.5 nm. Such an ultrathin interlayer should not be regarded as a thick blocking dielectric. Instead, photocarrier transfer across the ZnO/SiOx/Si interface can still occur through tunneling or tunneling assisted transport, while the interlayer may also improve interface quality [35, 36, 37]. This interpretation is consistent with the finite open circuit voltage and short circuit current observed across the full spectral range. The Si region exhibits well‐ordered lattice fringes, while the ZnO film appears dense and uniform across the field of view, with no obvious voids or extended defects.

A higher‐magnification TEM image of the ZnO layer alone (Figure 2h) resolves clear atomic‐scale lattice fringes, indicating that the film is highly crystalline over tens of nanometers and free of significant grain‐boundary contrast in the imaged area [22]. This high crystallinity is essential for stable pyroelectric and photoconductive response under repeated optical cycling. The corresponding energy dispersive spectroscopy (EDS) elemental maps in Figure 2i further verify the layer structure: the Zn (green) and O (magenta) signals are confined to the top film, whereas the Si (cyan) signal is localized in the underlying substrate, with minimal overlap between the three channels. The abrupt change in elemental contrast across the interface confirms that the ZnO layer is compositionally pure and that interdiffusion at the ZnO/Si junction is limited, providing a well‐defined platform for engineering the internal electric fields exploited in our in‐sensor feature‐extraction scheme.

The Zn 2p core‐level spectrum (Figure 2j) shows two well‐resolved peaks corresponding to the Zn 2p_3/2_ and Zn 2p_1/2_ components. The Zn 2p_3/2_ peak is centered near ≈1022 eV and the Zn 2p_1/2_ peak near ≈1045 eV, giving a spin–orbit splitting ΔBE ≈ 23 eV, which is characteristic of Zn^2+^ in ZnO rather than metallic Zn or other zinc compounds [24, 38]. The peaks are symmetric and do not exhibit additional shoulders at lower binding energy, indicating the absence of significant metallic Zn or sub‐oxide states and confirming that Zn is predominantly in the +2‐oxidation state throughout the film. The O 1s spectrum (Figure 2k) can be deconvoluted into two main components. The dominant peak at ≈530 eV is attributed to lattice oxygen (O_L_) in Zn–O bonds, consistent with stoichiometric ZnO [38]. A weaker, higher‐binding‐energy shoulder around ≈531–532 eV is commonly associated with oxygen in defect‐related states, such as surface hydroxyl/adsorbed species. The relatively high intensity of the lattice‐oxygen contribution compared to the defect‐related component indicates that the film is largely stoichiometric, while the controlled presence of oxygen‐related defects may contribute to the observed pyroelectric and photoconductive behavior. Together with the TEM/EDS analysis, these XPS results confirm that the active layer is a chemically well‐defined ZnO thin film suitable for reliable device operation.

Figure 2l compiles the pyroelectric response into a two‐dimensional map of peak *I*_E_ as a function of wavelength (vertical axis, 365–940 nm) and applied bias (horizontal axis, –40 to +40 mV). The color scale spans from ≈0.05 µA (dark blue) to ≈10.5 µA (dark red). At negative bias (–40 to –20 mV), the *I*_E_ spikes remain small in magnitude over the entire spectral range, typically below ≈0.05–1 µA even at the longest wavelengths. As the bias approaches 0 mV, the magnitude of the *I*_E_ gradually increases for visible–NIR wavelengths, while remaining weak in the UV, producing an oblique transition band (near‐white region). For forward bias above ≈+10 mV, the *I*_E_ response becomes strongly wavelength dependent. In the visible range (≈500–700 nm), the peak *I*_E_ rises to a few microamperes, whereas in the NIR (≥800 nm) it is further amplified, reaching up to ≈10 µA around 880–940 nm at +30–40 mV. In contrast, the UV wavelengths remain close to the low‐current (blue) regime across all biases. This bias–wavelength map shows that the device not only produces robust pyroelectric spikes over a broad spectrum, but also that their sign and amplitude can be systematically tuned by millivolt‐level biases in a wavelength‐selective manner—providing a rich, analog “fingerprint” that can be exploited for event‐driven, spectrally encoded sensing [11, 39, 40]

Figure 2m quantifies how both the steady *I*_P_ and *I*_E_ can be tuned by small external biases. At a fixed wavelength, the three *I*_E_ traces (–40, 0, and +40 mV) reveal that the *I*_E_ can be modulated over a wide range by only a few tens of millivolts: moving from –40 mV (grey) to 0 mV (red) and +40 mV (blue) progressively enhances the broadband response and especially boosts the NIR events, where *I*_E_ reaches values above ∼8–9 µA. The corresponding *I*_P_ spectra (green, magenta, and yellow) show that the steady photocurrent is also bias‐programmable: at –40 mV the response remains relatively small across the spectrum, whereas 0 V and +40 mV selectively amplify the visible–NIR component, with *I*_P_ approaching several microamperes near 900–950 nm. Importantly, the shapes of the *I*_E_ and *I*_P_ curves do not scale identically with bias, indicating that the same voltage setting can independently reweigh the *I*_E_ and quasi‐static channels. Thus, by choosing the lateral bias *V*_AI_, the device not only reconfigures its spectral sensitivity but also tunes the relative strength of *I*_P_ and *I*_E_, providing two coupled yet separately controllable analog features for downstream classification.

To elucidate the origin of the bias‐programmable self‐powered response, we simulated the spatial distribution of photogeneration and carrier collection in the Ag/ZnO/*n*‐Si/Ag vertical stack contacted by six top probes on the ZnO side (see Experimental Section for details). The central probe serves as the readout contact (*I*_out_), and its neighbors on each side act as tuning electrodes. In the binary bias patterns used in Figure 2n,o, “0” denotes 0 V and “1” denotes +100 mV applied to the corresponding probe (counted from left to right). Experimentally, the measured *I*_out_ (both *I*_E_ and *I*_P_) shows a clear dependence on applied bias. Figure 2n,o plot the normalized collected carrier density as a function of wavelength and depth for two representative patterns, 100001 and 001100, respectively. When only the outermost probes are biased (100001), the collection region remains confined near the surface over the full spectral range. In contrast, biasing the central pair of probes (001100) produces a substantially deeper collection region, particularly for wavelengths around 350–450 nm where the Si absorption length is comparable to the depletion width. This behavior is consistent with a scenario in which the external probes locally perturb the effective built‐in fields of the Ag/ZnO, ZnO/*n*‐Si and *n*‐Si/Ag junctions, thereby reshaping the collection probability of photogenerated carriers that drift/diffuse toward the readout contact. To capture this mechanism, we use a simplified two‐dimensional electrostatic/collection model that incorporates wavelength‐dependent absorption in ZnO (dominant in the UV) and *n*‐Si (visible–NIR) together with a phenomenological field‐dependent collection term.

**FIGURE 3:**
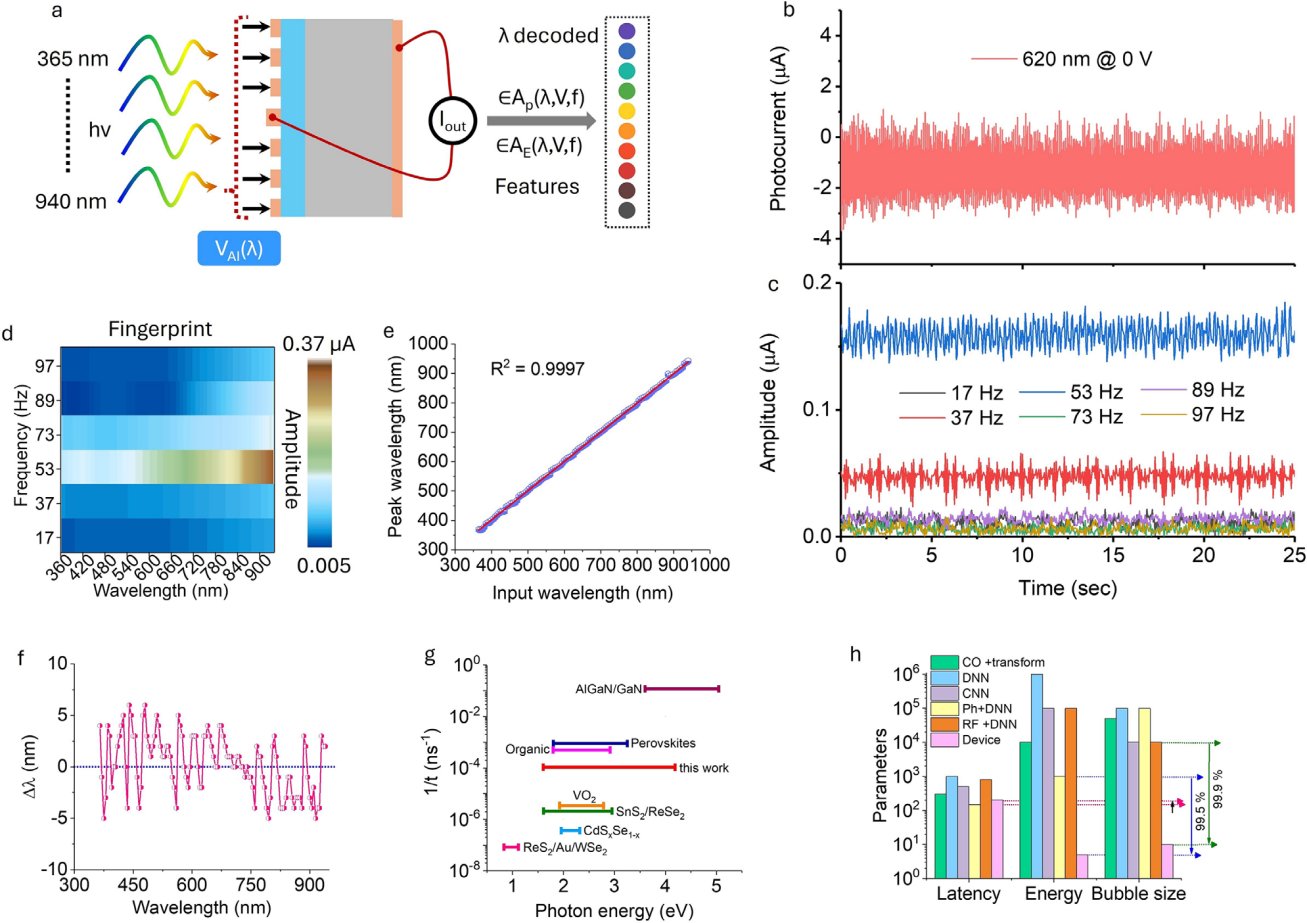
Frequency‐multiplexed photo–pyro fingerprinting, wavelength decoding and system‐level comparison. (a) Concept of the multi‐frequency in‐sensor classifier. Pulsed light (365–940 nm) illuminates the multi‐terminal Ag/ZnO/Si/Ag device while each top electrode is driven with a distinct small AC bias *V*_AI_(*f*). The resulting output current *I*_out_(*t*)contains a superposition of photocurrent and pyroelectric contributions that are demodulated at each drive frequency to yield photo and event amplitudes *A*_P_(λ, *V*, *f*)and *A*_E_(λ, *V*, *f*), which form an analog feature vector used for wavelength decoding. (b) Output current *I*_out_(*t*) at 620 nm and 0 V DC bias under simultaneous six‐tone excitation (17, 37, 53, 73, 89, and 97 Hz, 100 mV amplitude) and 50 Hz pulsed illumination (2 mW cm^−2^). (c) Lock‐in demodulation of panel (b), showing the extracted current amplitudes at each drive frequency. (d) Fingerprint map summarizing the demodulated amplitudes as a function of wavelength and frequency. (e) Decoded peak wavelength versus input wavelength obtained by matching the measured fingerprints to pre‐calibrated templates. Data falls tightly on the identity line, with a linear fit *R* ^2^ = 0.9997. f) Wavelength‐dependent decoding error, Δ*λ* = *λ*_decoded_ − *λ*_in_, measured across 10 randomly selected devices under different operating conditions. (g) Comparison of intrinsic response speed and spectral coverage of this work with representative neuromorphic/retinomorphic photodetectors. Our device occupies a previously unfilled region combining broadband operation with microsecond‐scale dynamics. (h) Normalized system‐level comparison of representative sensing pipelines in terms of latency, energy per classification, and effective computational complexity. The in‐sensor approach exhibits lower downstream energy cost and effective computational complexity while maintaining comparable end‐to‐end latency, highlighting the advantage of direct physical feature encoding inside the sensor. The latency, energy, and computational‐complexity comparison in (h) is evaluated for narrowband peak‐wavelength identification at the matched two‐point resolution of approximately 5 nm.

Following the tunability of with V_AI_, we moved forward with the utilization of our device for accurate color classification. Figure 3a schematically illustrates the working principle of our frequency‐multiplexed and wavelength classifier. Selective wavelength pulsed light in the range 365–940 nm impinges on the multi‐terminal Ag/ZnO/Si/Ag device, generating both a static photocurrent *I*_P_ and fast pyroelectric spikes *I*_E_. To capture these two contributions in a form that is highly discriminative in wavelength, each of the six top Ag electrodes is driven by a small AC bias at a distinct frequency while keeping the amplitude fixed at 100 mV: six distinct bias frequencies (17, 37, 53, 73, 89, and 97 Hz) are simultaneously applied to the electrode array (Figure S5, Supporting Information). These frequencies are selected to avoid their harmonics. These millivolt‐level tones modulate the local built‐in fields and are mixed by the device into a single output current *I*_out_(*t*) measured at the bottom contact. From *I*_out_(*t*), we numerically demodulate the response at each drive frequency to obtain the corresponding current amplitudes associated with the photocurrent plateau, *A*_P_(λ, *V*, *f*), and with the event‐driven pyroelectric spikes, *A*_E_(λ, *V*, *f*). The resulting six‐dimensional vectors {*A*_P_, *A*_E_}constitute a unique “fingerprint” for each wavelength, because the relative strength of photo and pyro components at the six frequencies depends sensitively on how the illumination wavelength reshapes the internal potential landscape. Although we use six electrodes and six frequencies here for clarity, the same framework is readily extendable to a larger number of terminals and to reconfigurable bias patterns, providing a scalable route to programmable in‐sensor feature extraction [41, 42]

The multifrequency demodulation and template matching steps serve only to read out and identify the fingerprint generated intrinsically by the self‐powered multiterminal device, rather than to impose spectral selectivity externally, because the wavelength‐dependent response space arises from the coupled photo and pyroelectric processes within the device itself. For wavelength decoding, the incident optical intensity was kept fixed for all measured wavelengths so that the extracted six channel fingerprints could be compared under identical excitation conditions, ensuring that the observed differences reflect the wavelength‐dependent redistribution of the mixed photo and pyroelectric response across the device rather than a trivial change in absolute signal level. In addition, because the pyroelectric contribution is driven by the instantaneous temperature change during light switching, slow ambient temperature drift or gradual cumulative heating does not by itself generate the sharp pyroelectric spikes used for fingerprint extraction. Temperature can nevertheless influence the mixed fingerprint by modifying the built‐in potentials and carrier transport across the Ag/ZnO, ZnO/Si, and Si/Ag junctions.

Figure 3b illustrates how the multifrequency drive encodes a wavelength‐specific fingerprint in the current output. The top panel shows the raw photocurrent *I*_out_(*t*) of the device at 620 nm under 0 V DC bias, when all six electrodes are driven simultaneously with 100 mV AC and the illumination is pulsed at 50 Hz (intensity = 2 mW cm^−2^). The waveform appears noisy but in fact consists of a deterministic superposition of the six drive tones (17, 37, 53, 73, 89, and 97 Hz) mixed with the photo–pyro response of the device to the 50 Hz light pulses. The lower panel (Figure 3c) shows the amplitudes extracted by sliding lock‐in demodulation at each drive frequency. For 620 nm, the largest response is obtained at 53 Hz, with a typical amplitude of ∼0.15 µA. This dominance arises because the 53 Hz tone lies closest to the 50 Hz optical modulation, so the electrical drive and the pyroelectric transients mix most strongly at this frequency. The remaining tones at 17, 37, 73, 89, and 97 Hz exhibit smaller but clearly nonzero amplitudes that reflect the wavelength‐dependent photo and pyroelectric response of the device sampled at each drive frequency rather than the physical position of the corresponding electrode (Figure S5, Supporting Information), confirming that each channel is set by its drive frequency and the intrinsic device response rather than by electrode location. Importantly, the full six‐dimensional amplitude vector changes with wavelength, which is the property exploited for decoding.

Figure 3d condenses the multifrequency response into a two‐dimensional “fingerprint” map, where each column corresponds to a wavelength (365–940 nm) and each row to one of the six drive frequencies (17–97 Hz). The color scale encodes the demodulated current amplitude, ranging from ≈0.005 µA (dark blue) to ≈0.37 µA (dark brown). At short wavelengths (360–420 nm) all six amplitudes remain small, typically below ≈0.02–0.03 µA, indicating that the lateral bias modulation has only a weak effect when absorption is confined near the top interface. As the wavelength increases into the visible–NIR region, the amplitudes grow and become strongly frequency‐selective: the 53 Hz channel, in particular, exhibits a nearly monotonic increase with wavelength, reaching ≈0.1–0.4 µA around 880–900 nm, while the neighboring frequencies remain at intermediate levels (few×10^−2^ µA) and the 17 Hz channel stays comparatively weak across the spectrum. Consequently, the six‐dimensional amplitude vector evolves in both magnitude and relative weighting as a function of wavelength, yielding a set of well‐separated, device‐intrinsic spectral fingerprints that we subsequently use as templates for wavelength decoding. When the optical modulation frequency was changed from 50 Hz to 40 and 80 Hz, the wavelength‐dependent evolution of the multifrequency response remained observable, although the relative channel amplitudes changed and the largest response shifted toward the electrical tone closest to the optical modulation frequencies (Figure S6, Supporting Information). These results show that the wavelength‐dependent device response persists under detuned optical modulation, while also demonstrating that operating‐frequency‐specific calibration is required. We therefore perform all wavelength decoding at a fixed optical modulation frequency of 50 Hz using templates acquired under the same condition.

To validate the wavelength decoding process, we examined the multifrequency fingerprint in greater detail in the 690 to 720 nm range (Figure S7, Supporting Information). The magnified fingerprint map and the extracted current amplitudes for two closely spaced wavelengths, 710 and 715 nm, show that even a 5 nm wavelength difference produces clear relative changes across several frequency channels. This confirms that the wavelength information is encoded in the combined multifrequency current pattern rather than in a single photocurrent value. For wavelength decoding, the demodulated amplitude vector of each measured signal was compared with a calibrated fingerprint library constructed from monochromatic sources with known peak wavelengths, and the best‐matched fingerprint was assigned as the decoded wavelength. Repeated measurements showed that the average absolute deviation between the decoded wavelength and the known peak wavelength remained below 3 nm. This metric reflects decoding accuracy for the peak wavelength of a monochromatic input rather than spectrometer‐like spectral resolution, because the present device is designed for electrically encoded wavelength identification and task‐level color sensing rather than full spectral reconstruction.

Dark and continuous‐illumination controls under the same multifrequency AC biasing further confirm the optoelectronic origin of the fingerprint (Figure S8, Supporting Information). In the dark, the demodulated current forms a wavelength‐independent per‐channel background that is removed by subtracting the dark demodulated amplitude vector, so the fingerprint differs from the electrical background across the six channels rather than being produced by it. Under continuous illumination the demodulated response is weak across the spectrum, because the pyroelectric contribution vanishes, whereas modulated illumination restores the full fingerprint. These controls exclude electrical crosstalk, capacitive coupling, and lock‐in demodulation as the source of the wavelength‐resolved response.

The achievable spectral resolution follows from the wavelength sensitivity of the demodulated fingerprint. With the six‐element amplitude vector A(*λ*) and its noise covariance Σ, the Cramér–Rao localization precision is $\sigma_{\lambda }={\left[{\left(\frac{\partial A}{\partial \lambda }\right)}^{T}\Sigma^{-1}\left(\frac{\partial A}{\partial \lambda }\right)\right]}^{-1/2}$ ≈ 2.5 nm, consistent with the mean absolute decoding error in Figure 3e, while the two‐point spectral resolution is *δλ* ≈ 2*σ*_λ_ ≈ 5 nm, consistent with the experimental separation of 710 and 715 nm in Figure S7. The value quoted below 3 nm is therefore the single‐peak localization precision rather than the two‐point resolution, and the device operates close to the information limit set by the fingerprint gradient.

The spectral resolution is obtained from the device's wavelength decision function. Decoding one monochromatic input scores the measured six‐element fingerprint against the calibrated library and returns a peaked decision spectrum whose width is set by the fingerprint gradient and the per‐channel noise. Superposing the decision spectra of two equal inputs resolves them at 5 nm separation and merges them at 2 nm (Figure S9, Supporting Information), giving a two‐point resolution *δλ* ≈ 5 nm equal to twice the 2.5 nm single‐peak localization accuracy quoted as below 3 nm and matching the measured 710 and 715 nm pair of Figure S7.

To further examine the robustness of the wavelength encoded fingerprints, we additionally measured the multi frequency response under varying light intensity, across multiple devices, and at different temperatures. The overall fingerprint pattern is preserved over illumination intensities from 0.4 to 2.0 mW cm^−2^ (Figure S10, Supporting Information), across 10 separately prepared devices (Figure S11, Supporting Information), and between 23°C and 40°C (Figure S12, Supporting Information), although the absolute amplitudes vary moderately with operating condition. Consistent with this picture, the temperature‐dependent measurements at 23°C and 40°C shown in Figure S12 in the Supporting Information confirm that the fingerprint remains clearly resolved at both temperatures, although the amplitude distribution shifts systematically with temperature. To further distinguish wavelength variation from intensity variation, additional wavelength dependent fingerprint measurements were carried out over a broad optical power range from 0.4 to 2.0 mW cm^−2^, and the corresponding maps in Figure S10 show that changing the optical power mainly rescales the absolute demodulated amplitudes, whereas the relative multifrequency fingerprint shape for a given wavelength remains substantially preserved. Additional measurements were performed on ten nominally identical devices to assess device‐to‐device reproducibility of the extracted multifrequency fingerprints, and the corresponding results are provided in Figure S11 in Supporting Information. This confirms that the decoding principle relies primarily on the preserved multi frequency fingerprint shape and wavelength specific channel distribution rather than on the absolute current magnitude alone. Together, these results indicate that the classification signatures are governed primarily by the relative multi frequency fingerprint structure rather than by a single absolute current value, supporting robustness against moderate source fluctuation, device to device variation, and temperature change within the present laboratory range. A dedicated long term drift study was not performed here, and systematic evaluation under extended operation remains an important subject for future work.

To quantify the fidelity of our fingerprint‐based wavelength decoding, we plot in Figure 3e the decoded wavelength as a function of the input wavelength over the full 365–940 nm range (see Experimental Section). For fingerprint measurements, monochromatic pulsed illumination was generated by electrically modulating the light source with an ESP32‐based driver. The wavelength‐dependent measurements were performed sequentially, and the decoding shown in Figure 3e therefore corresponds to the peak wavelength of the monochromatic source used in each measurement. In principle, broadband light sources can also be used when temporal modulation is applied to preserve the pyroelectric response. Each data point corresponds to a monochromatic illumination for which the measured multifrequency amplitude vector is compared against the calibrated fingerprints and the best‐matching template is selected. The points lie almost exactly on the identity line, indicating a nearly perfect one‐to‐one mapping between physical and decoded wavelength. A linear fit of λ_decoded_versus λ_in_yields: λ_decoded_ = (0.9937 ± 0.0001) λ_in_ + (4.50 ± 1.08) nm, with Pearson's correlation coefficient *r* = 0.99985 and *R* ^2^ = 0.9997. The slope very close to unity and the small intercept (≈4–5 nm) confirm that the decoding is essentially unbiased and highly linear across the entire spectral window. These results show that the multi‐tone photo–pyro fingerprints provide a robust, quasi‐analog mapping from wavelength space to feature space and back, enabling accurate wavelength readout without explicit spectral reconstruction or training a machine‐learning model.

Figure 3f analyses the decoding error in more detail by plotting Δλ = λ_decoded_ − λ_in_ as a function of input peak wavelength. The errors scatter symmetrically around zero over the entire 360–940 nm range, with no visible long‐range drift, consistent with the almost ideal slope and small intercept obtained from the linear fit in Figure 3e. In the UV–visible region (≈365–600 nm) the points show a slightly larger spread but still remain confined within a few nanometers of the zero line, whereas in the red–NIR region (≈700–940 nm) the fluctuations tighten further, reflecting the particularly robust fingerprints generated when absorption is dominated by the silicon junctions. Across the whole spectrum, Δλ stays well within ±5 nm and is typically only a few nanometers. This error distribution demonstrates that the multifrequency photo–pyro fingerprints enable stable, low‐bias wavelength decoding without systematic offsets, and that the residual fluctuations are dominated by local noise rather than by any fundamental limitation of the encoding scheme. Accordingly, the significance of the present system lies not only in reducing external computation, but also in shifting the decisive wavelength‐encoding step from software into the coupled device physics of the sensing element.

Figure 3g benchmarks the spectral range and intrinsic response speed (1/τ) of our device against various previously reported photodetectors. Conventional wide‐bandgap AlGaN/GaN devices offer very fast responses but are restricted to a narrow deep‐UV band at high photon energy. (42]. Organic and perovskite photodetectors extend operation into the visible, yet their characteristic times are typically slower, in the sub‐millisecond to millisecond regime. Phase‐transition or 2D‐material‐based devices (VO_2_, SnS_2_/ReSe_2_, CdS_x_Se_1‐x_, ReS_2_/Au/WSe_2_) often operate at even lower photon energies but with response times that orders of magnitude are slower still [8, 9, 10, 11, 14, 16, 17, 18, 39, 40, 41, 42]. In contrast, our approach spans a broad photon‐energy range covering the visible to near‐infrared and part of the UV, while maintaining a response rate 1/τ around 10^−4^ ns^−1^, corresponding to event times in the tens‐of‐microseconds regime [15]. This places our pyro‐phototronic feature‐extraction device in a previously unoccupied region of the performance space, combining broadband spectral operation with microsecond‐scale dynamics that are substantially faster than most existing photodetectors. While compact spectral identification has previously been demonstrated in electrically tunable van der Waals platforms [13, 21], the present device relies on a different physical mechanism, namely self‐powered retinomorphic pyro‐phototronic encoding through electrostatically balanced built‐in potentials and multifrequency bias modulation.

It is important to mention here that in Figure 3g, the time taken during the ML processes was not considered; however, this is an important factor for real‐time and energy efficient operations. Figure 3h provides a normalized system‐level comparison of representative wavelength‐sensing pipelines in terms of latency, energy per classification, and effective computational complexity. This panel is intended to illustrate the relative downstream burden required to obtain one final decision from the sensor output, rather than to serve as a strict hardware‐to‐hardware benchmark on a single optimized platform. Here, latency denotes the end‐to‐end decision time, energy per classification denotes the normalized sensing and decision cost per event, and bubble size is used as a normalized indicator of effective computational complexity. The relative reduction is evaluated as (*X*_ref_−*X*_proposed_)/*X*_ref_×100%, where *X*_ref_ and *X*_proposed_ are the normalized values of the reference and present schemes, respectively [27]

Relative to a photonic frontend + deep neural network (DNN) pipeline, our device operates with 50% higher latency (300 vs 200 ms), even though close. However, in return it cuts the energy per classification by ≈99.5%, from 1000 down to 5 (a 200 × reduction). At the representational level, the hardware is extremely compact: its effective bubble size is only 0.1% of that of a convolutional neural network (CNN) reconstructor (10 vs 10,000 parameters/features), corresponding to a 99.9% reduction in learned degrees of freedom while still enabling accurate wavelength decoding. Compared with heavier digital reconstructor pipelines (DNN/CNN and random‐filter RF), our in‐sensor scheme therefore occupies a distinctly favorable corner of the latency–energy–capacity space, where a modest latency overhead buys orders‐of‐magnitude gains in both energy efficiency and model compactness—highlighting the fundamental advantage of physical, in‐sensor feature extraction over conventional reconstruction‐plus‐DNN approaches [8, 9, 10, 11, 12, 13, 14, 15, 16, 17, 18, 39, 40, 41, 42]. This comparison is made for the matched task of narrowband peak‐wavelength identification at the two‐point resolution of ∼5 nm supported by the six‐harmonic fingerprint, so the reduced energy and computational cost reflect the fixed low‐dimensional readout rather than a coarser resolution (Table S2, Supporting Information).

**FIGURE 4:**
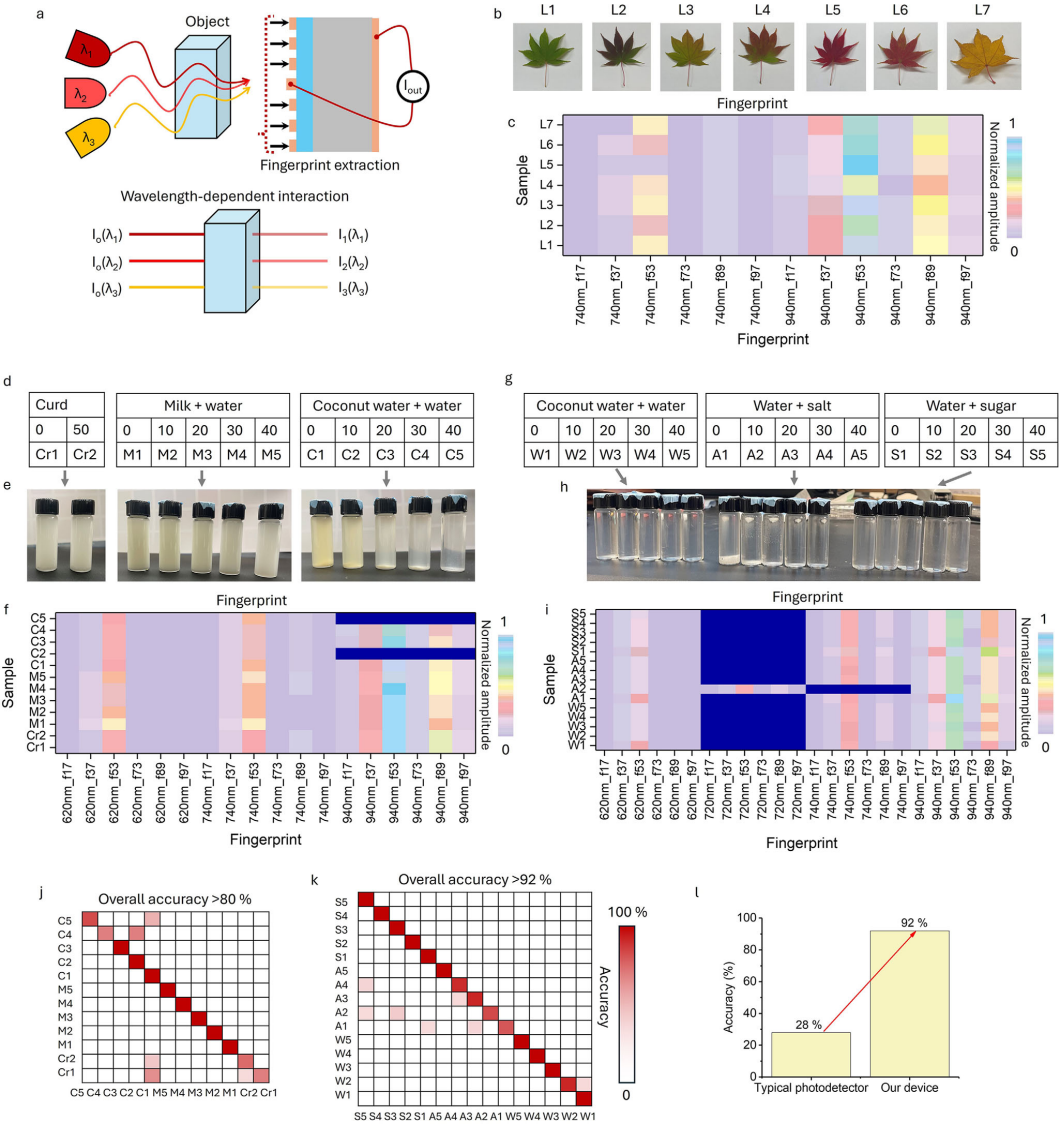
In‐sensor fingerprinting of biological and liquid samples and template‐based classification. (a) Conceptual illustration of object sensing with the multifrequency pyro‐phototronic device. Three representative wavelengths λ_1_,λ_2_,λ_3_illuminate an object (e.g., leaf or liquid). Their transmitted intensities *I*_0_(λ_i_) are modified by wavelength‐dependent absorption and scattering into *I*_1_(λ_i_), which are then converted by the multi‐terminal ZnO/Si detector into a wavelength‐ and sample‐specific electrical fingerprint via bias‐programmable photo–pyro feature extraction. (b) Photographs of maple leaves L1–L7, spanning a natural evolution from green through red to yellow. (c) Normalized 12‐dimensional fingerprints for the seven leaves, constructed from the multifrequency amplitudes at 740 and 940 nm. Each row corresponds to one leaf and each column to a wavelength–frequency channel; the systematic change in fingerprint pattern reflects differences in chlorophyll content and internal structure. (d) Composition table for dairy and beverage samples: curd (Cr1, Cr2), milk–water mixtures with 0%–40% added water (M1–M5), and coconut‐water–water mixtures with 0%–40% added water (C1–C5). (e) Representative photographs of the corresponding vials, showing only modest visual contrast between many samples. (f) Fingerprint map for the curd/milk/coconut‐water set, based on amplitudes at 620, 740, and 940 nm and six drive frequencies (18 channels in total). Each class and dilution level produces a distinct multiwavelength, multifrequency pattern, enabling separation of both composition and concentration. (g) Composition table for transparent‐liquid samples: coconut water diluted with water (W1–W5), pure water with added salt (A1–A5), and pure water with added sugar (S1–S5), each spanning 0%–40% solute or dilution. (h) Photographs of the transparent‐liquid vials, which are nearly indistinguishable to the naked eye. (i) Normalized 24‐dimensional fingerprints for the transparent‐liquid set, obtained at four wavelengths (620, 720, 740, and 940 nm) and six frequencies. The three series (W, A, S) occupy clearly distinct regions of this feature space, and the fingerprints evolve systematically with concentration. (j) Confusion matrix for template‐based classification of the curd/milk/coconut‐water fingerprints in panel (f). The main diagonal is strongly populated, with only minor off‐diagonal entries, giving an overall accuracy exceeding 80%. (k) Confusion matrix for the transparent‐liquid fingerprints in panel (i) Misclassifications is largely confined to adjacent concentration levels, yielding an overall accuracy above 92%. (l) Comparison of classification accuracy obtained using a typical transmittance‐based photodetector (28%) and our multifrequency pyrophototronic device (>92%) under the same template‐matching scheme.

As potential applications, we used our wavelength‐fingerprint sensor as spectroscopy‐inspired applications such as agriculture (Figure 4). Three narrowband sources at 620, 740, and 940 nm illuminate an object, for example, a leaf, a liquid sample, or another agricultural material—and the transmitted light is collected by the multi‐terminal Ag/ZnO/Si/Ag device [39]. Because each wavelength interacts differently with the object, according to its chemical composition (pigments, water content, dissolved solids, etc.), the transmitted intensities *I*_1_(λ_1_), *I*_2_(λ_2_), and *I*_3_(λ_3_) become wavelength‐ and sample dependent. These spectrally modified beams then impinge on the photodetector, where our multifrequency biasing scheme performs fingerprint extraction and converts the residual optical information at 620, 740, and 940 nm into distinct multi‐tone photo–pyro amplitude patterns. In this way, the object's wavelength‐dependent interaction is directly encoded into an electrical fingerprint at the sensor output, enabling compact classification and quantification of sample properties without full spectral reconstruction or external feature engineering.

Figure 4b,c demonstrates how the wavelength–fingerprint concept translates into a realistic agricultural sensing task. We collected seven maple leaves (named L1–L7) spanning a gradual color change from healthy green to dark red and finally yellow, corresponding to progressive depletion of chlorophyll and evolution of other pigments (Figure 4b). Each leaf was sequentially illuminated at 740 and 940 nm, and the transmitted light was analyzed by our multifrequency device to extract the photo–pyro amplitudes at the six drive frequencies. The resulting 12‐dimensional feature vectors $\left[A\left(740\;\mathrm{nm},f_{1\text{…}6}\right),\;A\left(940\;\mathrm{nm},f_{1\text{…}6}\right)\right]$ are depicted in Figure 4c as a fingerprint map. Even without any machine‐learning model, the fingerprints evolve systematically along the leaf series: channels associated with 740 nm, which probe chlorophyll absorption, vary strongly between green and senescent leaves, while the 940 nm channels, which are more sensitive to water content and scattering, exhibit a complementary modulation pattern. Each leaf thus produces a distinct, high‐contrast pattern across the 12 channels, despite all measurements being obtained from a single‐pixel device. This example illustrates that our in‐sensor feature extraction can capture subtle biochemical and structural differences in real biological samples and convert them directly into compact, wavelength‐resolved analog signatures suitable for downstream classification/quantification.

Figure 4d–f extends this concept from leaves to liquid food samples. We prepared three families of samples (Figure 4d): (i) curd with two different water mixture (named: Cr1 pure, Cr2 with 40% of water), (ii) milk progressively diluted with water from 0% to 40% (named: M1–M5), and (iii) coconut water diluted with water from 0% to 40% (named: C1–C5). Representative photographs in Figure 4e show only indirect visual differences between many of these samples, particularly among the various milk dilutions. Each sample was sequentially probed using the three wavelengths (620, 740, and 940 nm), and the transmitted light was analyzed by our device to extract the multifrequency photo–pyro amplitudes, yielding an 18‐dimensional fingerprint vector $\left[A\left(620\;\mathrm{nm},f_{1\text{…}6}\right),\;A\left(740\;\mathrm{nm},f_{1\text{…}6}\right),\;A\left(940\;\mathrm{nm},f_{1\text{…}6}\right)\right]$. The resulting fingerprints are visualized as a heat map in Figure 4f, where rows correspond to individual samples and columns to the wavelength–frequency channels. The curd samples (Cr1, Cr2) share a strong, characteristic response at 620 nm that clearly separates them from all milk and coconut‐water mixtures. Within the milk series (M1–M5), the amplitudes at 620 and 740 nm evolve systematically with added water, reflecting the dilution of fat and protein, whereas the 940 nm channels, which are more sensitive to water content, show a complementary trend. The coconut‐water series (C1–C5) occupies a distinct region of this feature space, with markedly different patterns at 740 and 940 nm compared to both curd and milk, and the high‐dilution samples (C4, C5) produce nearly saturated responses in specific 940 nm channels. Thus, each class (curd, milk, coconut water) and each dilution level within a class gives rise to a reproducible, high‐contrast multiwavelength fingerprint, obtained directly in the sensor without spectral reconstruction or digital feature engineering.

Figure 4g–i show that the same device can distinguish even visually transparent aqueous solutions and their concentration. We prepared three series of liquids (Figure 4g): coconut water progressively diluted with pure water (W1–W5), pure water with added NaCl (A1–A5), and pure water with added sucrose (S1–S5), each spanning 0%–40% dilution. As seen in the photographs (Figure 4h), the vials are nearly indistinguishable by eye. Each sample was then probed at three wavelengths (620, 740, and 940 nm), and the multifrequency photo–pyro amplitudes were extracted and normalized, yielding a 24‐dimensional fingerprint per sample. The resulting heat map (Figure 4i) reveals clear, class‐specific patterns: the diluted coconut water series (W1–W5) produces a characteristic evolution of the 620 and 940 nm channels with added water; the salt solutions (A1–A5) display a distinct signature dominated by a sharp change in the 720 nm channels at intermediate salinity; and the sugar solutions (S1–S5) occupy yet another region of the feature space, with a different balance of 740 and 940 nm responses. Thus, even for transparent liquids with subtle optical contrast, the device converts wavelength‐ and concentration‐dependent interactions into separable, high‐dimensional fingerprints that can be used for label‐free identification and quantification of ionic versus non‐ionic solutes. (39]

To show how well the analog fingerprints produced by our device support sample classification using a simple, model‐free template matcher, we quantify the results using the confusion matrix (Figure 4j,k). Figure 4j corresponds to the dairy/coconut‐water task of Figure 4d–f, where we jointly classify curd (Cr1, Cr2), milk–water mixtures (M1–M5), and coconut‐water–water mixtures (C1–C5) using their 18‐dimensional fingerprints. The main diagonal is uniformly dark red, with only a few lights off‐diagonal pixels, indicating that almost all samples are mapped to the correct class; the average accuracy over all categories exceeds 80%, even though neighboring mixtures (for example successive milk dilutions) are optically very similar. Figure 4k depicts the performance for the transparent‐liquids task of Figure 4g–i, where coconut‐water dilutions (W1–W5), salt solutions (A1–A5), and sugar solutions (S1–S5) are classified based on their 24‐dimensional fingerprints. Here the diagonal is even cleaner, with only weak off‐diagonal entries confined to adjacent concentrations within the same chemical family. The resulting overall accuracy exceeds 92%, demonstrating that the physically encoded multiwavelength, multifrequency signatures are sufficiently rich to distinguish not only the type of solute (ionic vs non‐ionic vs none) but also its concentration, using only a lightweight, template‐based decoder and no machine‐learning training. To verify this, we measured the transmitted signal using a conventional photodetector and applied a simple template‐matching scheme to the resulting data. As shown in Figure 4l, this approach yields a poor classification accuracy of only ∼28%, which is dramatically lower than our device, where the same template‐matching strategy achieves >92% accuracy.

Beyond class discrimination, the fingerprint provides continuous concentration calibration. For the milk and coconut‐water dilution series the dilution‐responsive 740 nm channel increases monotonically and approximately linearly with water content over 0% to 40%, with linear fits giving *R* ^2^ = 0.98 and 0.96 (Figure S13a). A leave‐one‐out test, refitting the calibration on four concentrations and predicting the fifth, recovers the held‐out interior concentrations with root‐mean‐square errors of 4.1% and 6.4% (Figure S13b). The Fisher‐information resolvable interval $\Delta c={\left[{\left(\frac{\partial A}{\partial c}\right)}^{T}\Sigma^{-1}\left(\frac{\partial A}{\partial c}\right)\right]}^{-1/2}$ ≈ 1.6% for milk and 2.9% for coconut water lies well below the 10% steps, establishing quantitative interpolation between the measured concentrations rather than coarse discrimination.

## 3 Conclusion

In conclusion, we have developed and experimentally validated a machine‐learning‐free, self‐powered broadband multiterminal pyro‐photodetector that performs direct wavelength encoding in hardware. Although implemented in a familiar Ag/ZnO/n‐Si material platform, the advance lies in the coupled operation of wavelength‐dependent built‐in field redistribution, pyroelectric transients, and multifrequency lateral biasing, which together generate a distinct joint photo‐ and pyroelectric electrical fingerprint for each wavelength. Through the careful coupling of optical absorption, charge transport, and electrostatic modulation within a single element—via electrostatically balanced built‐in potentials—our platform achieves purely electrical wavelength encoding less than 3 nm accuracy and a pyroelectric current rise‐time of ∼46 µs. This physically encoded readout is leveraged to demonstrate accurate color classification, plant health and pigment‐state sensing, and quantification of water adulteration (0%–40%) in milk, curd, coconut water, and salt/sugar solutions with >92% accuracy, all without invoking external machine‐learning pipelines. Taken together, these results establish a new paradigm of on‐chip, real‐time, energy‐efficient, and drift‐tolerant color recognition that circumvents the size–complexity trade‐offs of bulk optics and the computational overheads of digital inference. Our portable retinomorphic photodetector thus emerges as a general platform for nanometer‐precision spectral sensing across science, industry, and healthcare, and provides a blueprint for future neuromorphic and retinomorphic architectures in which physical device physics—not algorithmic post‐processing—perform the core tasks of sensing, encoding, and classification, as depicted in Table S1 (Supporting Information).

Although the present proof‐of‐concept system completes one full classification cycle in 300 ms, the underlying photodetector exhibits a pyroelectric current rise time of ∼46 µs. This difference indicates that the present bottleneck lies primarily in readout and signal processing rather than in the intrinsic device response. The results therefore highlight substantial room for future latency reduction through optimized electronics and real‐time signal processing. Under controlled laboratory conditions, the encoded fingerprints remain consistent under moderate variations in light intensity, across nominally identical devices, and over a limited temperature window, supporting the physical robustness of the encoding scheme. At the same time, the present pre‐calibrated templates should be regarded as temperature‐specific, and broader practical deployment will require temperature‐aware calibration or compensation, systematic evaluation under stronger environmental noise, larger source fluctuations, and long‐term drift, as well as continued efforts to reduce device‐to‐device variability, since measurable differences in absolute fingerprint amplitude still make device‐specific calibration appropriate at this stage. In addition, repeated measurements performed over a period of three months under the same laboratory conditions did not show any significant change in the overall multi‐frequency fingerprint pattern, although a systematic long‐term drift study under broader environmental variations was not performed in the present work. Importantly, although the current prototype relies on manually applied contacts and external readout electronics, the ZnO/Si heterostructure and multiterminal architecture remain compatible with future planar integration and faster dedicated readout schemes.

## 4 Experimental Section

### 4.1 Device Fabrication and Characterization

Zinc oxide thin films were grown by plasma‐enhanced atomic layer deposition (PEALD; Nexusbe, Korea). A thickness of 100 nm was targeted, using a substrate temperature of 250°C with diethylzinc (DEZ) as the Zn precursor and oxygen as the co‐reactant. One ALD cycle consisted of a 0.3 s DEZ pulse, a 2 s N_2_ purge, a 3 s O_2_ pulse, a 1 s O_2_ plasma step, and a 3 s purge. These steps were carried out at a working pressure of 1 Torr with an RF plasma power of 100 W. The experimentally determined growth rate was 0.14 nm per cycle, so approximately 714 cycles were required to obtain a 100 nm film (714 × 0.14 nm ≈ 99.96 nm). With a nominal cycle duration of 9.3 s, the total deposition time for a 100 nm layer was ∼110 min. The top and bottom electrodes were prepared using silver paste as a proof‐of‐concept contacting method for rapid device validation.

Angle‐resolved X‐ray photoelectron spectroscopy (AR‐XPS) measurements were conducted on a Thermo Fisher Scientific NEXSA system equipped with a monochromate Al Kα radiation source (hν = 1486.6 eV). All binding energies were referenced to the adventitious carbon C 1s signal at 284.8 eV. Cross‐sectional microstructural analysis was performed by high‐resolution transmission electron microscopy (HR‐TEM) using a JEOL JEM‐2100F, with lamellae prepared by focused ion beam (FIB) milling.

### 4.2 Measurements

To probe the device behavior under combined electrical and optical excitation, linear sweep voltammetry and chronoamperometry were carried out using a Zive SP1 potentiostat/galvanostat (ZIVELAB). Time‐resolved current–voltage signals were acquired from the ZnO/Si pyro‐phototronic detector under pulsed illumination at multiple wavelengths. Each measurement sequence consisted of six simultaneous low‐amplitude sinusoidal bias tones (17, 37, 53, 73, 89, and 97 Hz) applied across the electrode array through a custom multi‐channel DAC interface. For the multifrequency biasing and read‐out of the Ag/ZnO/*n*‐Si multiterminal photodetector, we built a microcontroller‐based platform centered on an ESP32 development board (top left). The ESP32 communicates via the I^2^C bus (SDA/SCL) with a TCA9548A I^2^C multiplexer, which expands the bus to address multiple MCP4725 12‐bit DAC modules (bottom left, red boards; represented schematically in the dashed blue box in Figure S14, Supporting Information). Each DAC is programmed to generate a small‐amplitude sinusoidal voltage (typically 100 millivolts peak) at a distinct frequency and to apply this bias to one of the Ag top electrodes of the Ag/ZnO/n‐Si device through a custom probe (top right photograph in Figure S14, Supporting Information). In this way, different electrodes are driven at different AC tones while sharing a common reference. The bottom Ag contact of the device is connected to a commercial potential (Zive SP1 potentiostat/galvanostat) configured in transimpedance mode, which records the total output current *I*_out_ containing the superimposed photo‐ and pyro‐current components from all excited terminals. All electronic modules share a common ground and are powered from the ESP32 supply, and the ESP32 firmware controls the waveform amplitude, offset, and frequency of each DAC channel while the potentiostat output is acquired by the measurement computer for subsequent demodulation and analysis.

Because the device operates under self‐powered conditions, electrically induced self‐heating is expected to be minimal in the present measurements, and the observed temperature dependence is attributed primarily to temperature‐dependent evolution of the junction potential and the coupled photo and pyroelectric response.

Optical measurements were performed using monochromatic light sources, including narrowband LEDs and laser diodes, depending on the wavelength range. For the measurements in Figure 2b, the current‐voltage characteristics were recorded by a standard voltage sweep under continuous monochromatic illumination, so these data correspond to the steady‐state photocurrent response rather than the transient pyroelectric response. In contrast, the transient and wavelength fingerprint measurements were carried out under pulsed illumination generated by electrical modulation of the light source using an ESP32 based driver. In the fingerprint measurement, the illumination frequency was 50 Hz with an incident intensity of 2 mW cm^−2^. In the present study, the optical excitation was quantified by the incident intensity at the device rather than by independently calibrated single pulse energy.

To exclude an electrode‐position artifact, we performed a defined electrode and frequency reassignment control under spatially uniform illumination over the active device area. The six driving frequencies were rearranged from 17, 73, 53, GND, 37, 89, 97 Hz to 53, 37, 17, GND, 73, 97, 89 Hz, moving the 53 Hz tone from the electrode adjacent to the grounded reference to the outermost electrode, while the device geometry and the illumination condition were kept unchanged (Figure S5, Supporting Information). The wavelength‐dependent multifrequency fingerprint remained unchanged within experimental error after reassignment, confirming that the fingerprint is governed by the drive frequency and the intrinsic optoelectronic response of the device rather than by electrode position.

The consistent multifrequency fingerprints observed across multiple devices indicate that the operating principle is reproducible despite the manual contacting process. In future scalable implementations, these manually applied contacts can be replaced by lithographically patterned metal electrodes on the same ZnO/Si platform without changing the underlying device architecture. In the present proof‐of‐concept system, feature encoding occurs in the sensing device itself, whereas the AC bias generation, signal demodulation, and final classification are implemented using external electronics and software.

### 4.3 Simulation of Photocurrent Generation With Bias

The effect of the lateral probe biases on the self‐powered response was analyzed using a simplified two‐dimensional model implemented in MATLAB. The device was represented as a planar Ag/ZnO/*n*‐Si/Ag stack with a 100 nm ZnO layer on top of a *n*‐Si substrate. Seven circular top contacts (diameter ∼100 µm) were placed on the ZnO surface to mimic the experimental probes: one central contact defined as the grounded and bottom electrode was considered as a readout electrode (*I*_out_, fixed at 0 V). Three contacts on each side that could be biased at either 0 or +100 mV. The illumination was assumed to be normal incidence from the ZnO side. Wavelength‐dependent optical generation *G*(λ, *z*) was treated semi‐analytically: strong absorption in ZnO was assumed in the UV, while visible–NIR photons were taken to transmit through ZnO and be absorbed in Si according to effective absorption coefficients. The Ag/ZnO, ZnO/n‐Si and n‐Si/Ag interfaces were described by fixed “effective built‐in” potential drops, chosen to be consistent with typical Schottky/heterojunction band offsets; explicit band diagrams, space‐charge regions and recombination kinetics were not solved self‐consistently. A phenomenological collection probability *P*(λ, *z*)was then defined, increasing with the magnitude and favourable sign of this local field and decaying with depth according to assumed drift/diffusion lengths in ZnO and Si. Maps of *P*(λ, *z*) were calculated for different bias configurations, and the differences (e.g., ∣*P*_biased_ − *P*_all 0V_∣) were used to visualise how biased probes near versus far from ground electrode modify the effective collection of photocarriers generated in the UV‐sensitive ZnO and in the visible–NIR‐sensitive Si. This modelling framework is intentionally minimal: it treats ZnO and Si as layered media with effective built‐in fields and does not perform a full Poisson–drift–diffusion solution with explicit doping, interface states or trap‐assisted recombination. Consequently, the results are interpreted as qualitative, physically motivated illustrations of the spatially selective influence of the biased probes, rather than quantitative fits to the measured absolute photocurrent.

### 4.4 Amplitude Extraction for Different Frequencies

All analysis was performed using MATLAB. Each recorded file contained the current–time waveform for one sample and wavelength. After dark‐current subtraction and baseline detrending, a sliding lock‐in algorithm was used to demodulate the signal at each of the six drive frequencies. The lock‐in process isolated the tone‐specific amplitude envelope *A*_f_(*t*), effectively separating the photo‐ and pyro‐current contributions into distinct frequency channels. Averaging *A*_f_(*t*) overtime yielded a stable six‐dimensional amplitude vector for that measurement. For samples measured at multiple wavelengths, the amplitude vectors were concatenated across all (λ, *f*) combinations, forming a unique multi‐wavelength, multi‐tone fingerprint *A*_λ, f_for each run. All fingerprints from repeated runs were compiled into a feature matrix, where each row corresponded to one run and each column to a specific wavelength–frequency pair.

## Conflicts of Interest

The authors declare no conflicts of interest.

## Supporting information




**Supporting File**: adma74160‐sup‐0001‐SuppMat.docx.

## Data Availability

The data that support the findings of this study are available from the corresponding author upon reasonable request.
