# Supplementary material for: Electrically Coded Retinomorphic Spectrophotodetector
Source: Adv Mater. 2026 Jul 17;38(48):e74160. doi: 10.1002/adma.74160 (PMC13508704; doi:10.1002/adma.74160)
Supplement: Supplementary file 1 [file ADMA-38-e74160-s001.docx]

Supporting Information

**Electrically Coded Retinomorphic Spectrophotodetector**

Mohit Kumar,^1,2,*^ Hyunmin Dang,^2^ and Hyungtak Seo^1,2,*^

Department of Energy Systems Research, Ajou University, Suwon, 16499, Republic of Korea,

Department of Materials Science and Engineering, Ajou University, Suwon, 16499, Republic of Korea,

Email: [hseo@ajou.ac.kr](mailto:hseo@ajou.ac.kr) (H.S.), mohitiopb@ajou.ac.kr (M.K.)

**Table S1**. Comparison of representative pyro-phototronic and related multifunctional photodetectors in terms of response time, application scope, and color-sensing capability.

| S.N. | Device structure | Response time | Applications | Color sensing | Ref. |
| --- | --- | --- | --- | --- | --- |
| 1 | Cl-rich 2D/3D interface | 176 ps | LiDAR | … | 1 |
| 2 | BTO/GaN | 0.35 s | … | … | 2 |
| 3 | NiO/ZnO | Not reported | Pyro-photovoltaic | … | 3 |
| 4 | BaTiO3 film | Not reported | Imaging | … | 4 |
| 5 | p-NiO/n-ZnO | 8.9 µs | … | … | 5 |
| 6 | ZnTe/ZnO | 62 µs | … | … | 6 |
| 7 | p-Si/n-ZnO | 1.2 ms | … | … | 7 |
| 8 | ZnO/Ag | 3.5 ms | … | … | 8 |
| 9 | ZnO/CuO/Au | 12 ms | … | … | 9 |
| 10 | Cs_2_PbI_2_Cl_2_ perovskite | 18 µs | logic gate | … | 10 |
| 11 | CsAg_2_I_3_/GaN | 16 µs | Key management | … | 11 |
| 12 | β-Ga_2_O_3_ | 0.2 µs | logic gate | … | 12 |
| 13 | RbPb_2_Cl_5_ | 7 ms | … | … | 13 |
| 14 | Ag/ZnO/Si | ~46 µs | Plant health,  pigment-state sensing, water quantification in milk, curd, coconut  water, and salt/sugar solutions | color classification | This work |

**Figure S1**. Cross-sectional TEM image of the Ag/ZnO/n-Si device, showing a ≈100 nm-thick ZnO layer (measured 102.5 nm) on the Si substrate (scale bar: 20 nm).

**Figure S2**. Energy band diagram of the ZnO/n-Si heterojunction under equilibrium, showing the relative electron affinities (χ(ZnO)=4.35 eV, χ(Si)=4.05 eV), bandgaps (E_g,ZnO_=3.3 eV, E_g,Si_=1.12 eV), and the resulting conduction- and valence-band offsets (ΔE_C_ ≈ 0.3 eV and ΔE_V_ ≈ 2.48 eV).

**Figure S3**. Schematic illustration of the pyrophototronic response of the Ag/ZnO junction under different illumination conditions. a) Under dark, the c-axis–oriented ZnO layer exhibits the built-in band bending at the Ag/ZnO contact. b) When a light pulse is turned on, rapid heating generates pyroelectric polarization along the ZnO c-axis, inducing opposite bound charges at the two ends, enhancing the interfacial electric field and driving a transient pyroelectric current spike. c) Under continuous illumination, the temperature and polarization reach a quasi-steady state and only the steady photocarrier current remains. d) When the light pulse is switched off, cooling reverses the pyroelectric polarization, inverting the interfacial field and producing an oppositely directed current spike.

**Figure S4**. Time-resolved photocurrent response of the Ag/ZnO/n-Si device under pulsed illumination at 740 nm (2 mW cm^-2^). a) Conventional operation at a small reverse bias of –40 mV produces rectangular photocurrent pulses with a characteristic rise/decay time of ~150 μs. b) Near open-circuit conditions (≈30 mV), the same optical stimulus generates sharp pyroelectric current spikes with a much shorter response time of ~46 μs, highlighting the fast, spike-like operation.

**Figure S5**. Electrode and frequency assignment control for the multiterminal Ag/ZnO/n-Si pyro-phototronic detector under spatially uniform illumination. a) Six top electrodes carrying 17, 73, 53, GND, 37, 89, and 97 Hz from left to right around the grounded reference, the combined photo and pyroelectric generation and recombination (G+R) being read as a single multiplexed current at the bottom contact. b) Wavelength-dependent multi-frequency fingerprint measured in the original configuration shown in (a), reproducing the main-text fingerprint (Figure 3d). c) Reassigned configuration, 53, 37, 17, GND, 73, 97, and 89 Hz. d) Wavelength-dependent multi-frequency fingerprint measured in the reassigned configuration. The fingerprint remains the same within experimental error as in the original assignment (Figure 3d), with the 53 Hz channel still dominant and brightening toward the near infrared, confirming that the fingerprint follows the drive frequency and the intrinsic device response rather than electrode position.

**Figure S6**. Optical modulation frequency control. Wavelength-dependent multi-frequency photocurrent amplitude maps recorded under a) 40 Hz and b) 80 Hz optical modulation, for comparison with the 50 Hz map of Figure 3d. The wavelength-dependent structure is preserved at both frequencies, the active channels retaining their rise toward the near-infrared, while the channel of largest amplitude shifts to the electrical tone nearest the optical modulation frequency, moving to 37 Hz at 40 Hz optical modulation and to 73 Hz at 80 Hz. This optical-frequency dependence identifies the readout as a light-driven photo-pyro heterodyne process and confirms the transient pyroelectric origin of the fingerprint rather than an instrumental near-resonance or leakage artifact.

**Figure S7**. Experimental validation of wavelength discrimination from the multi-frequency electrical fingerprint. a) Full wavelength-dependent fingerprint constructed from the demodulated current amplitudes at different driving frequencies, showing the characteristic electrical encoding pattern used for wavelength decoding (Figure 3d in the main manuscript). b) Magnified view of the fingerprint in the 690 to 720 nm region. The dashed lines indicate 710 and 715 nm, showing that even a 5 nm difference produces clearly distinguishable changes across multiple frequency channels. c) Extracted current amplitudes at 17, 37, 53, 73, 89, and 97 Hz for 710 and 715 nm. The relative amplitude changes at several frequencies confirm that wavelength identification is determined by matching the full multi-frequency fingerprint rather than by a single photocurrent value, thereby supporting the high wavelength decoding accuracy reported in the manuscript.

**Figure S8**. Dark and continuous-illumination controls under the same multi-frequency AC biasing. (a) Dark demodulated amplitude at each electrical channel. (b) Demodulated amplitude map under continuous illumination without optical modulation, weak across the spectrum relative to the modulated fingerprint of Figure 3d. Neither control reproduces the modulated fingerprint, confirming that the wavelength-resolved fingerprint arises from the genuine light-driven photo and pyroelectric response rather than from electrical crosstalk, capacitive coupling, and lock-in artifacts.

**Figure S9**. Spectral resolving capability of the device. a) Single-peak localization accuracy from the calibrated Figure 3e sweep. The absolute decoding error |decoded − input| at each of the 116 wavelengths from 365 to 940 nm scatters about a mean absolute value of 2.5 nm (MAE, dashed line), the localization accuracy reported as below 3 nm, with the upper line at twice this value, 5 nm. To ensure the best achievable accuracy, this measurement was performed under controlled conditions. b) Two-point resolving test built from the wavelength decision function. Each monochromatic input produces a decision spectrum obtained by scoring its measured six-element fingerprint against the calibrated library, and the decision spectra of two equal inputs separated by 8, 5, and 2 nm are superposed. The two maxima are clearly resolved at 8 nm, marginally resolved at 5 nm, and merged at 2 nm, giving a two-point resolution δλ ≈ 5 nm consistent with approximately twice the 2.5 nm localization precision and consistent with the measured 710 and 715 nm pair of Figure S6. Here the 8 nm and 2 nm separations are bracketing trials, not resolution values, and the intermediate 5 nm is the two-point resolution.

**Figure S10**. Intensity-dependent wavelength fingerprints of the multiterminal device measured over an optical power range from 0.4 to 2.0 mW cm^-^². Each panel shows the demodulated current amplitude as a function of wavelength and frequency at a different incident intensity. As optical power decreases, the absolute amplitude is reduced, but the characteristic multi-frequency fingerprint structure remains substantially preserved across the measured range, including the wavelength-dependent frequency selectivity and the dominant response channels. These results indicate that moderate intensity variation mainly rescales the signal magnitude, whereas the wavelength-specific fingerprint pattern remains distinguishable.

To further distinguish wavelength variation from intensity variation, additional measurements were carried out over a broad optical power range from 0.4 to 2.0 mW cm⁻², and the corresponding wavelength-dependent fingerprint maps are provided in Figure S7 in the Supporting Information. These data show that changing the optical power mainly rescales the absolute demodulated amplitudes, whereas the relative multi-frequency fingerprint shape for a given wavelength remains substantially preserved. Thus, decoding is governed primarily by the wavelength-specific channel distribution rather than by the absolute current magnitude alone.

**Figure S11**. Device-to-device reproducibility of the multi-frequency wavelength fingerprints measured from ten nominally identical Ag/ZnO/*n*-Si/Ag devices under identical experimental conditions. Each heat map shows the demodulated current amplitude as a function of wavelength and modulation frequency using a common color scale. All devices preserve the same characteristic fingerprint structure, including the dominant response near 53 Hz and the consistent wavelength-dependent evolution of the channel amplitudes, confirming that the wavelength-encoding mechanism is reproducible across independently prepared samples. Minor differences in absolute amplitude are observed among devices, which are attributed to normal device-to-device variation in local field distribution, built-in potential balance, and manual contact formation. The white color in these heat maps corresponds to absence of measurable data. The lower-right panel summarizes the device-to-device amplitude variability, showing that the spread remains low for most devices. These results indicate that the hardware fingerprint-generation mechanism is reproducible, while the present proof-of-concept implementation still benefits from device-specific calibration for optimal decoding accuracy.

**Figure S12**. Temperature-dependent wavelength fingerprints measured at 23 °C and 40 °C. The characteristic multi-frequency spectral fingerprint remains clearly resolved at both temperatures, confirming that the wavelength-encoding principle is preserved under moderate thermal variation. At the same time, the relative amplitude distribution shifts systematically with temperature, indicating that the pre-calibrated templates are temperature-specific rather than strictly temperature-invariant.

**Figure S13**. Quantitative concentration calibration. (a) Calibration curves. The normalized amplitude of the dilution-responsive 740 nm channel, f89 for milk and f73 for coconut water, is plotted against added water content for the five-level dilution series, with linear fits giving R^2^ = 0.98 (milk) and 0.96 (coconut water). (b) Leave-one-out validation. Each concentration is removed in turn, the calibration is refit on the remaining four, and the held-out water content is predicted from its amplitude, giving predicted-versus-actual values with RMSE = 4.1% (milk) and 6.4% (coconut water). The dashed line is the identity.

**Figure S14**. Schematic and photographs of the multi-frequency driving and readout setup. An ESP32 microcontroller controls a TCA9548A I^2^C multiplexer and four MCP4725 DAC modules that apply distinct AC biases to the six top electrodes of the Ag/ZnO/n-Si device through a probe card, while the total output current I_out_ is measured by a potentiostat/current amplifier.

**Table S2**. Matched-resolution comparison of representative miniature computational spectrometers with the present device. The reconstructive systems reach finer resolution by increasing the channel count and inverting an ill-conditioned sensing matrix, so their decoding cost grows with resolution, whereas the present device decodes a fixed six-element fingerprint by nearest-neighbour matching at O(6N) independent of resolution within the regime the six harmonics support.

**Table S2.** Matched-resolution comparison of representative miniature computational spectrometers with the present device. The reconstructive systems reach finer resolution by increasing the channel count and inverting sensing matrix, so their decoding cost grows with resolution, whereas the present device decodes a fixed six-element fingerprint by nearest-neighbour matching at O(6N) independent of resolution within the regime the six harmonics support.

| **Platform** | **Resolution** | **Channels (N)** | **Reconstruction** | **Decoding cost** | **Ref.** |
| --- | --- | --- | --- | --- | --- |
| Colloidal quantum-dot filter array | ~1 nm, 390 to 690 nm | 195 filters | regularized matrix inversion | grows with N | [14] |
| Single nanowire | ~7 to 8.5 nm, 510 to 710 nm | 30 to 38 units | compressive reconstruction | grows with N | [15] |
| Disordered photonic chip | ~0.75 nm at 1500 nm | hundreds of speckle channels | transmission-matrix inversion | grows with N | [16] |
| Tunable van der Waals junction | ~3 nm, 405 to 845 nm | single detector, gate sweep | responsivity-matrix reconstruction | grows with sweep points | [17] |
| ZnO/Si | ~5 nm two-point, 365 to 940 nm | 6 frequency channels | nearest-neighbour template match, no inversion | fixed O(6N) | This work |

**References**.

[1] L. Min, H. Sun, L. Guo, Y. Zhou, M. Wang, F. Cao, L. Li, *Adv. Mater*. **2024**, *36*, 2400279

[2] Y. Zhang, J. Chen, L. Zhu, Z. L. Wang, *Nano Lett*. **2021**, 21, 20, 8808–8816

[3] M. Patel, H.-H. Park, P. Bhatnagar, N. Kumar, J. Lee and J. Kim, *Nat Commun* **2024**, 15, 3466.

[4] N. Ma, K. Zhang, Y. Yang, *Adv. Mater*. **2017**, *29*, 1703694

[5] A. K. Rana, M. Kumar, D.-K. Ban, C.-P. Wong, J. Yi, J. Kim, *Adv. Electron. Mater*. **2019**, 5, 1900438

[6] D. You, C. Xu, W. Zhang, J. Zhao, F. Qin, Z. Shi, *Nano Energy*, **2019**, *62*, 310–318

[7] Z. Wang, R. Yu, X. Wang, W. Wu, Z. L. Wang, *Adv. Mater*. **2016**, *28*, 6880–6886

[8] Y. Wang, L. Zhu, Y. Feng, Z. Wang, Z. L. Wang, *Adv. Funct. Mater*. **2019**, *29*, 1807111

[9] Q. Li, J. Huang, J. Meng, Z. Li, *Adv. Optical Mater*. **2022**, *10*, 2102468

[10] Y. Zhao, S. Jiao, S. Yang, Y. Nie, Y. Han, J. He, C. Tao, D. Wang, S. Gao, J. Wang, L. Zhao, *Adv. Mater*. **2025**, *37*, 2414649

[11] Y. Zhai, S. Wang, P. Guo, S. Feng, P. Wan, J. Tang, C. Kan, H. Li, D. Shi, M. Jiang, *Advanced Science*, **2026**; 0:e74896

[12] P. Dang, T. Zhang, Z. Liu, S. Sun, Y. Wang, F. Zhang, K. Wang, X. Pan, B. Lu, L. Zhu, Z. Ye, J. Jiang, *Adv. Funct. Mater*. **2025**, *35*, 2504294

[13] D. Nasipuri, S. Karmakar, A. H. Sarkar, N. Goyal, S. Banerjee, R. Das, N. Ravishankar, K. D. M. Rao, N. Pradhan, *Adv. Mater*. **2026**, *38*, e10226

[14] J. Bao, M. Bawendi, A colloidal quantum dot spectrometer. Nature, **2015**, 523, 67–70. https://doi.org/10.1038/nature14576.

[15] Yang *et al*., Science, 2019, 365, 6457, 1017-1020, DOI: 10.1126/science.aax8814.

[16] B. Redding, S. Liew, R. Sarma, *et al*. Compact spectrometer based on a disordered photonic chip. Nature Photon **2013**, 7, 746–751. https://doi.org/10.1038/nphoton.2013.190

[17] Yoon et al., Science, 2022, 378, 296–299, https://doi.org/10.1126/science.add8544
